# Evaluation of solid tumor response to sequential treatment cycles via a new computational hybrid approach

**DOI:** 10.1038/s41598-021-00989-x

**Published:** 2021-11-02

**Authors:** Farshad Moradi Kashkooli, M. Soltani

**Affiliations:** 1 Department of Mechanical Engineering, K. N. Toosi University of Technology, Tehran, Iran; 2 Department of Electrical and Computer Engineering, University of Waterloo, Waterloo, ON Canada; 3 Cancer Biology Research Center, Cancer Institute of Iran, Tehran University of Medical Sciences, Tehran, Iran; 4 Advanced Bioengineering Initiative Center, Computational Medicine Center, K. N. Toosi University of Technology, Tehran, Iran

**Keywords:** Cancer models, Chemotherapy, Computational models, Tumour heterogeneity

## Abstract

The development of an in silico approach that evaluates and identifies appropriate treatment protocols for individuals could help grow personalized treatment and increase cancer patient lifespans. With this motivation, the present study introduces a novel approach for sequential treatment cycles based on simultaneously examining drug delivery, tumor growth, and chemotherapy efficacy. This model incorporates the physical conditions of tumor geometry, including tumor, capillary network, and normal tissue assuming real circumstances, as well as the intravascular and interstitial fluid flow, drug concentration, chemotherapy efficacy, and tumor recurrence. Three treatment approaches—maximum tolerated dose (MTD), metronomic chemotherapy (MC), and chemo-switching (CS)—as well as different chemotherapy schedules are investigated on a real tumor geometry extracted from image. Additionally, a sensitivity analysis of effective parameters of drug is carried out to evaluate the potential of using different other drugs in cancer treatment. The main findings are: (i) CS, MC, and MTD have the best performance in reducing tumor cells, respectively; (ii) multiple doses raise the efficacy of drugs that have slower clearance, higher diffusivity, and lower to medium binding affinities; (iii) the suggested approach to eradicating tumors is to reduce their cells to a predetermined rate through chemotherapy and then apply adjunct therapy.

## Introduction

The term “personalized medicine” was coined to describe the set of methods capable of administering the right drug for the right disease to the right patient. In oncology, this term encompasses a wide range of fields and techniques promising to tailor treatment to a specific patient’s cancer. Many properties of cancer are dynamic and patient-dependent, and evolve over time as well as in response to the timing and type of treatment. This fluidity makes the development of effective therapies difficult and complex. Moreover, lack of an individualized treatment plan for every patient, incorporating their own physiological parameters, leads to inconsistent responses to therapy and increases the risk of death, so better treatment options remain urgently needed. Precision medicine is deemed more beneficial than one-size-fits-all approaches^1^. Employing mathematical models and simulations that are capable of numerically describing and predicting the relations among drug exposure/pharmacokinetics (PK), drug effects/pharmacodynamics (PD), and disease evolution, is widely regarded an aid to making correct decisions about drugs, justifying personalized treatment, and addressing its failures^2–5^. Consequently, cancer researchers are now greatly interested in the personalization of cancer treatment through such models. Numerical and mathematical models offer a pain-free, quick, and cost-effective way of testing various potential drugs, situations and strategies along with other hypotheses.

Prediction of an effective dose regimen for chemotherapy is crucial to maximizing treatment efficacy. When new drugs are examined, dosing regimens are calculated later in the assessment process, during in vivo testing and the first phase of clinical trials^6^. This delay may cause problems since drugs with promising efficacy in vitro may not be efficacious in preclinical animal testing. The correct regimen may be capable of improving efficacy in vivo. One of the mechanisms influencing the efficacy in living systems is transport to and within tumor tissue^7^. An optimal treatment plan obtained by numerical modeling can overcome undesirable transport characteristics. In the absence of predictive models, dose-schedule determination processes first involve in vivo testing used to specify the lethal dose and the efficacy of potential drugs^6^. Effective drugs with controllable side effects are transferred to clinical trials. One of the main goals of conducting phase I trials is to specify the MTD, determined by gradually increasing the dose until dose-limiting toxicities are observed^8,9^. The suggested dose for phase II trials is determined at the MTD or a dose lower than that. With the passage of time, different chemotherapy approaches are designed according to drug dosage and its levels of toxicity, including MTD—densified high dose chemotherapy, MC—frequent low-dose chemotherapy (LDC), and adaptive therapy—varying doses and schedules of chemotherapy^10^. MC^11–13^, known as a form of multi-targeted therapy, and adaptive therapy^14,15^ have emerged as two possible alternative dosing schedule approaches to enhancing response rates while decreasing toxicities, since they modulate the dose and frequency of cytotoxics administration to control disease progression instead of eradicating it at all cost^10^. Recently, interest has been growing in evaluating these chemotherapy approaches by means of both clinical and mathematical methods^16–22^. Most of the previously-published mathematical models use systemic pharmacokinetics and kinetics of cell-death for evaluating drug delivery efficacy, but few involve both the spatio-temporal drug transport in tissue scale and tumor microenvironment (TME) heterogeneity, implying that most of the literature studies have used PK/PD models, which is just temporal, instead of full spatio-temporal models. These comprehensive studies have considered TME heterogeneity (e.g., interstitial fluid flow, microvascular network, elevated interstitial fluid pressure (IFP), inefficient lymphatic system, to name a few) and also employed spatial–temporal representations of tumor tissue to mathematically model drug transport in the TME^23–29^. Desirable transport properties of drug are crucial for efficient treatment of solid tumor^30,31^. Even with favorable properties against cells, drugs can still fail in vivo as a result of inefficient tissue-scale transport. The importance of transport properties arises from the physiology of TME. The cell-death rate depends on the local drug concentration^32^. Drugs unable to efficiently penetrate the tissue are not capable of clearing regions far from the vascular network although they can kill cells in a culture^33^. Additionally, the characteristics of a drug—diffusivity, associating rate, and systemic clearance rate—determine its capability to penetrate solid tumors^34,35^. Additionally, there have been many computational attempts to examine the effects of different drug parameters on the efficacy of drug delivery^23,32,35,36^, but their impact on successive treatment cycles have not yet been investigated.

In this study, the great potential of mathematical modeling and computational oncology is investigated for evaluating long-term response of treatment. A new approach is introduced as part of a solution to provide an efficient model for sequential cycles of cancer treatment towards the personalization of treatment based on the characteristics of each patient. To the best of the author’s knowledge based on the literature review and S.1 to S.4 sections in supporting file, this study is the first to consider the details of tumor growth, tissue transport, drug delivery, and treatment, simultaneously. Angiogenesis after each cycle of treatment may actually be different and heterogeneity in the spatial distribution of blood and lymphatic microvessels may happen, implying that structures of microvascular network and lymphatic drainage may change after each treatment cycle. Not only angiogenesis, but also tumor geometry would be different after each cycle of treatment^37,38^. Importantly, what makes the presented in silico hybrid approach stand out is that it solves the multiscale modeling problem by coupling tissue-scale with cellular-scale while considering long-term response of treatments. The main contribution of this study is methodological, and it is significant to recall possible shortcomings of the literature calibration we have opted for. In fact, previous studies have separately addressed interstitial fluid flow and drug delivery in solid tumors in a single cycle; however, connecting drug delivery and tumor growth in several cycles of treatment to identify better treatment protocols has not yet been investigated. To this aim, a case study is considered as a phase-Ι feasibility study of proposed treatment modeling to achieve a more-efficient and-realistic approach to evaluating treatment schedules. A set of theoretical drugs with various rates of diffusivity, binding affinity, and clearance can be evaluated by this treatment model. The effectiveness of each drug, measured by the fraction of killed cells (FKCs), can be calculated at various schedules. Treatment schedules are examined by changing the number and amount of doses. Treatment efficacies and general observations have then been extracted from these calculations.

## Results

In this study, an effort has been made to address important questions: “What is the advantage of alternative chemotherapy dosing strategies (LDC and CS) compared to a conventional one (MTD)?”; “How can different chemotherapy plans affect the treatment results?”, and “What is the result of the parameter study of drug transport properties on treatment outcomes?”. In the following, the results are provided in three sections for answering these questions.

### Chemotherapy approaches

“Conventional” here means administering chemotherapy at or near the MTD with drug-free breaks in between treatments to allow patients to recover from chemotherapy toxicities. This study has led us to provide novel theoretical models that support alternative methods to chemotherapy administration, i.e., MC and adaptive therapy. The CS regimen, a novel treatment modality that works between high and low doses of chemotherapy, has recently been introduced based on the use of MC after MTD chemotherapy.

**Figure 1:**
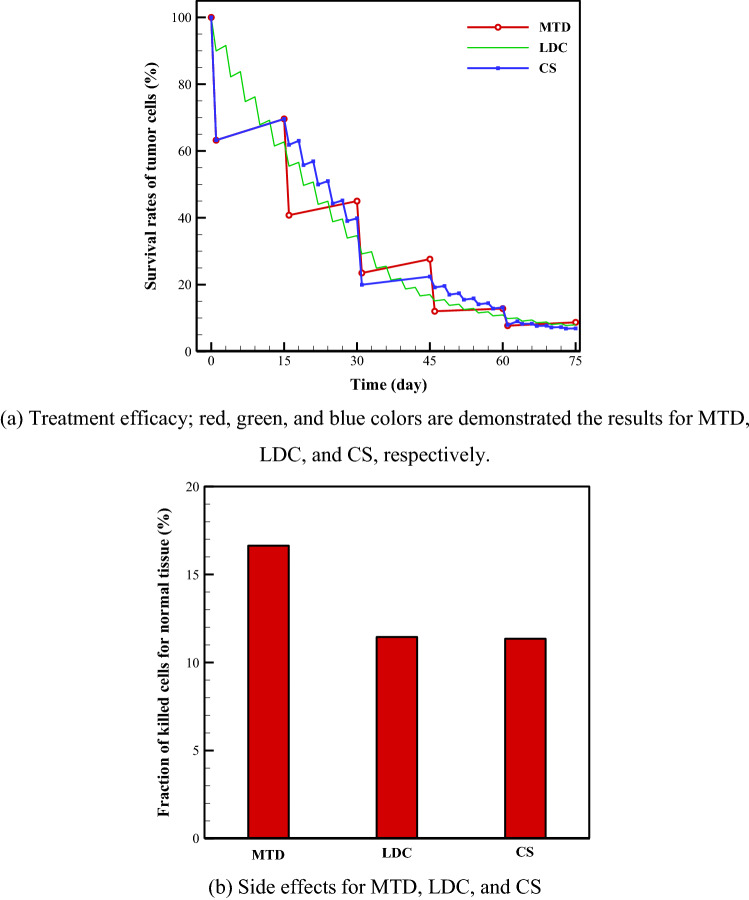
Survival rate of tumor cells and also side effects for different treatment approaches including MTD, LDC, and CS. These treatment approaches are defined as: (i) injected dose = *C*_*p*_ and interval between treatments = 0.5-month (for MTD); (ii) injected dose = 0.2*C*_*p*_, and interval between treatments = 0.1-month (for LDC); and (iii) injected dose of *C*_*p*_ and a 0.5-month break and then five administrations of 0.2*C*_*p*_ and a 0.1-month break (for CS). It should be noted that side effect is a secondary unwanted effect that occurs due to drug therapy. Here, side effects are defined as the impact of chemotherapeutic drug on the cells of healthy tissue around the tumor.

A comparison between the results of different chemotherapy administration approaches—MTD, LDS, and CS—are shown in Fig. 1. Cell-killing in a tumor suggests an important benefit for different drug regimens, but it may also result in serious side effects during interactions with the normal tissue surrounding the tumor or in different organs. Therefore, a therapeutic index is ordinarily employed for comparison between various approaches according to balanced quantification between both the side effects and efficacy of treatment in killing tumor cells (TCs). The plans considered are: *C*_*p*_ and a 0.5-month break between administrations for MTD, 0.2*C*_*p*_ and a 0.1-month break between administrations for LDC, and combinations of these two methods (i.e., *C*_*p*_ and a 0.5-month break and then five administrations of 0.2*C*_*p*_ and a 0.1-month break, and then repeating this period) for CS. A drug-free break is considered between the two successive cycles as recovery phase or drug holiday. The survival rates of TCs after 75 days are 8.7%, 8.0%, and 6.9% for MTD, LDC, and CS, respectively. The side effects of these three chemotherapy administration approaches are 16.6%, 11.5%, and 11.4%, respectively. These results demonstrated that MC or LDC and also CS will have considerable advantages over existing MTD regimens. It is also obvious that while the side effects of CS and LDC are almost the same, the treatment efficacy of CS is 1.1% higher than that of LDC. Although this percentage does not demonstrate a large difference between these two approaches, if this percentage is converted to the number of cells, it indicates a significant number of cells. As a trade-off between the maximum killing of TCs and minimum side effects introduces the best treatment method; so, CS, by having a 93.1% treatment efficacy and 11.4% side effects, is the best option among three different chemotherapy approaches investigated in this study.

### Chemotherapy schedules

**Figure 2:**
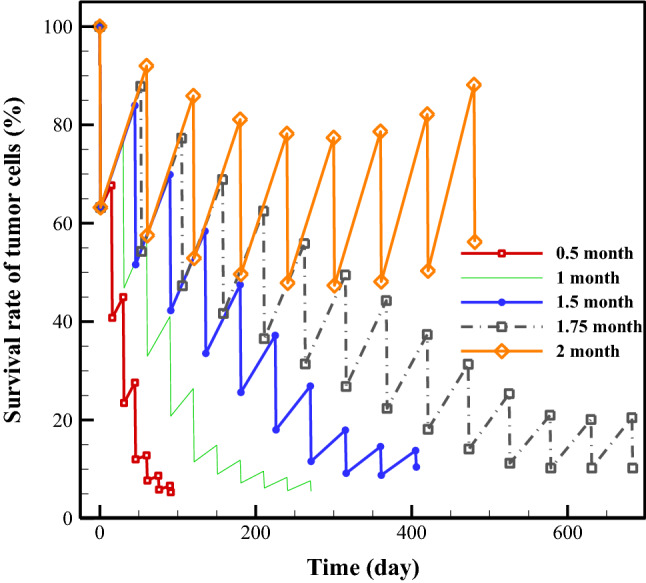
Survival rate of tumor cells for different times between successive treatments. Assuming the same injected dose (*C*_*p*_), the responses of TCs under five treatment plans with variable timing, including 0.5-month, 1-month, 1.5-month, 1.75-month, and 2-month, are examined here. Red, green, blue, gray, and orange colors are demonstrated the results for break timings of 0.5-month, 1-month, 1.5-month, 1.75-month, and 2-month, respectively.

Chemotherapy plans are usually characterized by three important parameters: timing, dosage, and drug type. In the present study, the drug doxorubicin (DOX) is considered in all investigations, and the effects of timing and dose are examined. First, assuming the same injected dose, the responses of TCs under five treatment plans with variable timing, including 0.5-month, 1-month, 1.5-month, 1.75-month, and 2-month, are evaluated. The survival rates of TCs are illustrated in Fig. 2 for the plans of interest. Results demonstrate that the rate of TC survival decreases after each treatment after tumor regrowth.

For the first treatment plan (i.e., starting at the baseline state), the effects of the 1st, 2nd, and 3rd cycles of treatment on the reduction of the TCs are approximately 36.78%, 26.93%, and 21.52%, respectively. This rate is 15.59%, 5.12%, 2.85%, and 1.33% for the 4th to 7th cycles of treatment, respectively. Tumor regrowth between the 1st and 2nd cycles of treatment, between the 2nd and 3rd cycles, and up to the end of the 7th cycle are calculated to be 4.48%, 4.23%, 3.13%, 1.79%, 1.01%, 0.8%, respectively. At the last treatment cycle, the TCs regrowth is almost 0.8%, while the therapeutic efficacy is approximately 1.33%, which means that using chemotherapy for this tumor size is not the best choice due to the side effects, so an adjuvant therapy is suggested at this stage. Overall, after seven treatment cycles, 5.32% of the initial TCs remain.

A similar trend is shown for the other treatment plans. However, the first scenario proves more efficient at killing TCs than the other scenarios. The rates of surviving TCs are 5.23%, 5.54%, 10.45%, 10.26%, and 56.12% with respectively 0.5-month, 1-month, 1.5-month, 1.75-month, and 2-month breaks between injections. Moreover, some plans (those with 1.5-month, 1.75-month, and 2-month breaks) are not efficient after a certain time, since the treatment cannot decrease the TCs because their rate of recurrence between two successive treatments is greater than their rate of decreasing. It has been demonstrated that the last stage of the 1.75-month plan and the last three stages of the 2-month plan have detrimental effects instead of beneficial ones in reducing TCs. Thus, the best decision in these circumstances is probably to use an adjuvant therapy (e.g., radiotherapy or surgery). In general, the timing or break between chemotherapy sessions is a very effective parameter on treatment efficacy. It should be mentioned that in the investigation of timing, since the injected dose is the same, the side effects are only dependent on the number of treatments.

**Figure 3:**
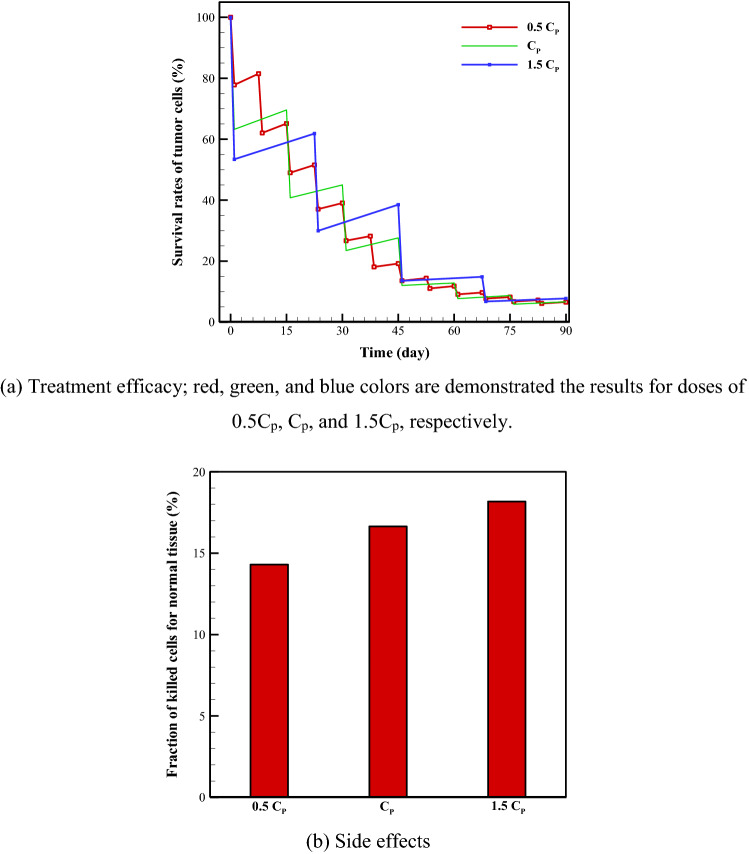
Survival rate of tumor cells and also side effects after 90 days for three different treatment scenarios to examine dose effect. These treatment plans are as: (i) injected dose = 0.5*C*_*p*_, number of treatments = 12, and interval between treatments = 0.25-month; (ii) injected dose = *C*_*p*_, number of treatments = 6, and interval between treatments = 0.5-month; and (iii) injected dose = 1.5*C*_*p*_, number of treatments = 4, and interval between treatments = 0.75-month.

As illustrated in Fig. 3, three treatment scenarios for evaluating dose effect were also compared based on the analysis of surviving rates of TCs. In the first treatment plan, the injected drug dosage is considered to be *C*_*p*_ and the total treatment number is six, with a 0.5-month interval between each two sequential treatments. With the second treatment plan, the amount of drug injected into the blood vessel is halved to 0.5*C*_*p*_ and twelve treatments are administered with 0.25-month breaks in between. In the last plan, the injected drug dosage is increased to 1.5*C*_*p*_ and the total treatment number is four, with a 0.75-month break between each two successive treatments. The resulting rates of survival in tumor and normal tissue cells after 90 days with these plans can be seen in Fig. 3.

Even though the dose for the first program is twice that of the second one and appears to be more toxic for patients, it does not result in a higher rate of killed TCs. The rates of surviving TCs are 6.6%, 6.7%, and 7.8% for 0.5*C*_*p*_, *C*_*p*_, and 1.5*C*_*p*_, respectively. In addition to success in killing cancer cells, significant attention must be paid to side effects on normal tissue during treatment. Hence, the FKCs in normal tissue are also examined. As shown in Fig. 3b, the FKCs of normal tissue are approximately 14.3%, 16.6%, and 18.2% after the entire treatment protocol for 0.5*C*_*p*_, *C*_*p*_, and 1.5*C*_*p*_, respectively. Based on these results, in addition to having more efficacy in killing TCs, the second scenario (0.5*C*_*p*_) is less damaging to normal tissue than the other scenarios.

### Parameter study on drug transport properties

**Figure 4:**
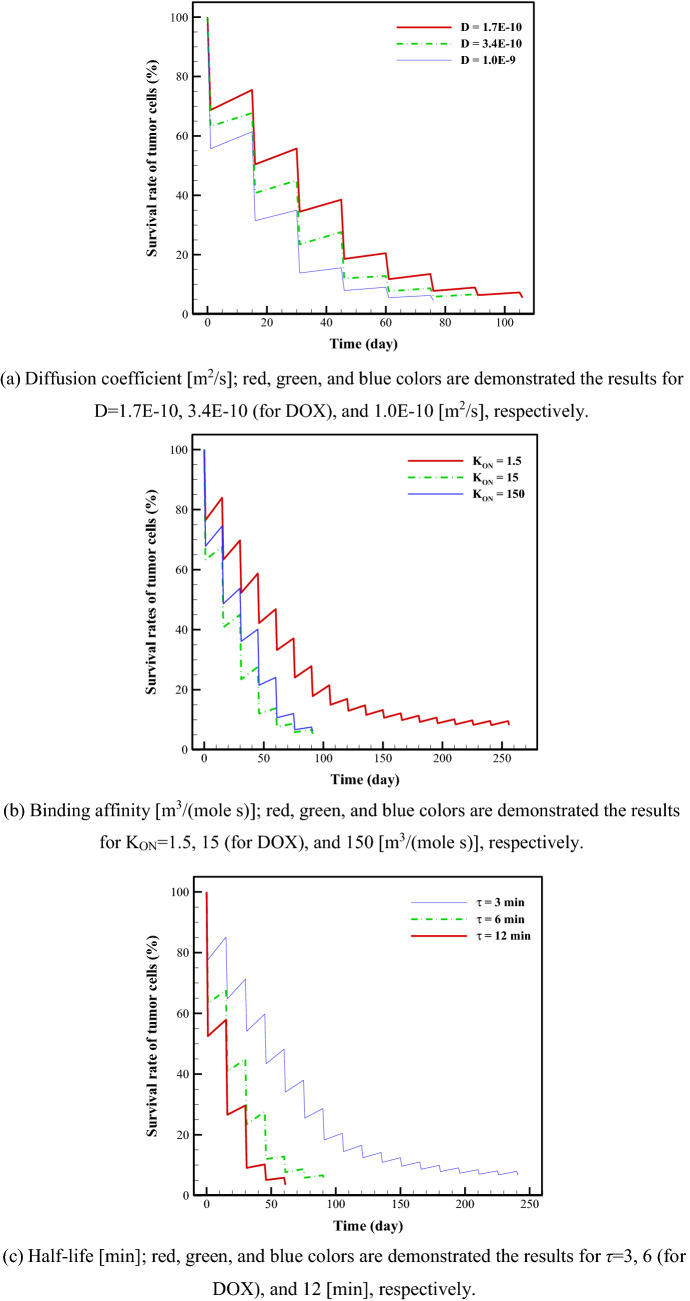
Survival rate of tumor cells for different (**a**) diffusion coefficients, (**b**) binding affinities, (**c**) half-lives. Parameter study of these three properties is performed to examine their impacts on drug delivery and treatment. To this aim, a baseline state is investigated with the same treatment plan for the different states considered, and then the values for the different parameters are changed. The baseline state is related to drug DOX and the other parameters are related to theoretical chemotherapeutic drugs. These parameter values are extracted from the measured values for DOX, as they have not been measured for most other clinical chemotherapy drugs. The ranges for different parameters were created to encompass most possible physical values.

The present study considers three important parameters—diffusion, binding affinity of drugs to cells, and drug half-life—to predict the influence of drug transport parameters on treatment results. Parameter study of these three properties has been carried out to investigate their effects on drug delivery and treatment. To this aim, a baseline state is considered with the same treatment plan for the different states considered, and then the values for the various parameters are changed. These parameter values are extracted from those for DOX, as they have not been measured for most other chemotherapy drugs. The effects of this change are demonstrated in terms of the survival rates of TCs over time. Multiple parameters are kept constant for all theoretical drugs and their amounts are matched to those of DOX. Figure 4 indicates the survival rate of TCs for different diffusion coefficients, binding affinities, and half-lives of drugs in plasma.

The survival rates of TCs have been evaluated for different diffusion coefficients after eight treatments with 0.5-month breaks between (Fig. 4a). The range of diffusivities is selected to be greater than the range from small to fast metabolite drugs to incorporate most possible chemotherapeutic drugs. Results for survival rates of TCs are 5.5%, 5.3%, and 4.6% for 1.7E−10 [m^2^/s] (50% decrease in baseline state value), 3.4E−10 [m^2^/s] (the baseline state), and 10E−10 [m^2^/s] (about 200% increase in baseline state value), respectively. It is obvious that increasing the diffusion coefficient enhances the treatment outcomes. At a slower diffusivity, the drug can barely enter the tissue, while at a higher diffusivity, more drug enters the tissue, so the drug concentration increases and higher rates of TCs will eventually be killed.

The effects of the binding affinity of ligands of drug to cell-surface receptors on treatment efficacy are shown in Fig. 4b. Actually, binding affinity is one of the adjustable properties of drugs, and may range from high to low. Here, three different binding affinity values are examined, and then survival rates of TCs are obtained. Enhancing the binding affinity from 1.5 [m^3^/(mole s)] (state 1) to 15 [m^3^/(mole s)] (state 2) and then 150 [m^3^/(mole s)] (state 3), the survival rate of TCs decreases from 11.4 to 5.3%, and then slightly increases to 6.2%. In other words, when binding affinity increases, first the FKCs increases and then decreases. Thus, the values of survival rates of TCs for intermediate values of binding affinity have the lowest amount and suggests the best treatment results (Fig. 4b). The numbers of treatments in states 1, 2, and 3 are 18, 7, and 7, respectively. In a tumor, rapid drug diffusion and high rate of drug binding compete with each other. Hence, non-uniform drug delivery and high binding affinity prevent homogeneous drug distribution and decrease a drug’s ability to reduce TCs. Two conditions must be met for efficient drug delivery: (i) the tumor must be exposed to the free drug as much as possible, (ii) the free drug must bind rapidly to prevent itself being washed out of the tumor. It has been demonstrated in the literature that a balanced value between high and low drug affinities can solve the problems of drug distribution and internalization^32,36^. In the present study, variations in the FKCs with binding affinity have a similar trend to that in Moradi Kashkooli et al.^32^ and Stylianopoulos et al.^36^ for DOX, which attests to the accuracy of the current study’s calculations.

The effects of drug half-life on treatment efficacy are shown in Fig. 4c, considering three different half-lives: 3 [min] (state 1), 6 [min] (state 2), and 12 [min] (state 3). It is demonstrated that the survival rates of TCs are 6.7%, 5.3%, and 3.5%, respectively. To achieve these percentages requires 18, 7, and 5 treatments, respectively, implying that drugs with long-clearance properties have better treatment efficacy than faster-clearance ones.

## Discussion

Considering the growing number of patients suffering cancer worldwide, plus the lack of methods for appropriate diagnosis and prediction of tumor growth, the high mortality associated with this disease will not decrease soon. In addition, the lack of treatment schedules tailored specifically for each patient and based on the patient’s own physiological parameters leads to inconsistent responses to treatment, an inefficiency that increases the risk of death from the disease. These factors have recently prompted a great deal of interest in researchers towards personalized medicine in cancer. To this aim, theoretical models of human tumors have helped to develop new chemotherapy treatment scenarios and identify better drug regimens. The primary models considered tumors as a simple collection of homogenous, exponentially-growing cancer cells. In contrast, subsequent models should take into consideration tumor-biology complexities, such as different levels of heterogeneity, and the influence of TME, so as to develop computer-based personalized protocols for the administration of chemotherapy. The development of a mathematical model that identifies adequate treatment regimens for individuals could help development of personalized therapies and enhance patient lifespans.

Previous researches in this field have separately addressed interstitial fluid flow and drug delivery in solid tumors in a single cycle, but connecting drug delivery and tumor growth in many cycles of treatment to characterize better treatment regimens has still not been investigated. In the present study, the potential of multi-scale spatio-temporal computational modeling is employed to evaluate long-term treatment response. Following this path, a novel methodology is introduced to provide a useful model for successive cancer treatment cycles based on the characteristics of each patient. Actually, the main idea of the presented methodology is adopted from the nature of tumor treatment and a great deal of attempts has been made to consider different details as much as possible. After applying a single treatment, the tumor shrinks to a smaller size with a new structure of microvascular network. Then, in the drug-free break between two consecutive treatments, tumor size increases in terms of the number of cells. This process will continue until the end of the treatment cycles administered for the patient. This study is the first to consider the details of tumor growth, interstitial fluid flow in tissue, drug transport, and treatment, simultaneously. Presented study can also calculate the IFP, drug exchange dynamics, distribution of various drug concentrations—free, bound, and internalized—and FKCs in tumor and normal tissues. Such a model can be used for quantifying therapeutic factors. Drug delivery modeling is carried out based on the drug transport modeling in solid tumor, which was developed by Baxter and Jain^39–41^, Wang et al.^42^, and Soltani et al.^31,43^. Subsequently, treatment efficacy is calculated, based on the empirical formula for different drugs described in S.4 in the Supplementary File, using the internalized drug concentration. According to the obtained value for efficacy, the percentage of tumor shrinkage is calculated. Finally, to prove the effectiveness of this methodology, a case study is taken into account to assess different treatment schedules.

Surgery and radiotherapy are currently the most common direct treatment methods used for the eradication of solid tumors. However, when cancer reaches the metastasis phase, cancer cells have spread from the initial tumor to other sites in the body, a systemic treatment such as chemotherapy is needed. Chemotherapy acts as a double-edged sword, eliminating cancer cells on the one hand, while destroying normal cells on the other hand. Due to the creation of drug resistance during cancer chemotherapy, eradicating cancer cells by hitting them with higher drug dosages as soon as possible has been suggested. On the other hand, high drug doses will result in excessive toxicity. The complicated dependency and interaction of these different aspects complicate the development of optimum treatment plans. Recently, the use of new approaches like MC and CS have been suggested because of their benefits in tumor treatment and lower side effects^22^. As continuous drug injection is clinically impossible owing to the very harmful side effects on normal cells, it is necessary to assess the efficacy of MTD, LDC, and CS methods comparatively. Despite promising preliminary results showed in different studies, only limited data has been available for a long time regarding the right dose of the different drugs to be used in the metronomic administration^44^. In many ways, development in MC and CS allows for the utilization of computational oncology at the bedside, since the optimization of an MC or CS regimen is largely achievable only with the help of modeling before clinical trials^16,45^. Based on the results of the current study, CS is the best approach among three different chemotherapy methods. West and Newton^19^ have also suggested the use of LDC instead of MTD in cancer treatment. Their justification is that the reduction of TCs is more sensitive to variations of dose density than variations of dose concentration, particularly for tumors that grow faster. Incorporating a large number of low doses in MC contrasts with the traditional “more is better” dosing approach used in MTD approach^46^. On the other hand, MC, as a monotherapy, has not been able to provide convincing results in clinical trials^20,21^. Till now, it could be used in just some phase III cases of breast cancer, head and neck tumors, colorectal cancer, and hepatocellular carcinoma^21^. Its combination with other drugs like Bevacizumab and Pazopanib, other therapeutic approaches such as radiotherapy, and/or encapsulation of chemotherapeutic agents in nanoparticles have been shown to potentiate treatment outcomes^20,22^. Single large doses (i.e., MTD) could be deemed necessary in cases where the dose windows are small, but might be less efficient for new targeted drugs with lower toxicity^30^. Our simulations demonstrate that the MC protocol is more effective than the more classical MTD protocol in eradicating solid tumors. This result has good consistency with the modeling results of Benzekry and Hahnfeldt^47^, Guiraldello et al.^48^, Terterov et al.^49^. In addition, despite the very promising results of CS method in the present study, there exists no certain clinical evidence to support its high efficiency so far^21^. Some clinical studies showed that a long-term exposure to one or more chemotherapeutic agents and deprivation of others, introducing CS or break periods of MTD with MC, may increase treatment efficacy^50^. This phenomenon is also called 4D (drug-driven dependency/deprivation) effect^51^. André et al.^52^ concluded that TCs become dependent on chemotherapy agents during long-term exposures and sudden replacement or withdrawal therapy may result in death of TCs. In all patients, toxicity level is of concern and the use of intermittent MC schedules might decrease toxicity while maintaining effectivity^50^. Some clinical results^53–55^ have also reported a successful combination of MC and other treatment approaches.

An ideal treatment plan consists of timing chemotherapy sessions correctly, determining the appropriate drug combinations, and setting the drug-dose levels based on pre-determined goals^46,56^. Although several clinical efforts have been made to determine efficient and reliable chemotherapy treatment plans, all involved many limitations because of the high cost, long testing time, and difficulty of the various trials and procedures. As many authors have pointed out, further refinement of the use and administration of chemotherapy requires special attention to accurate models and also numerical solutions, because clinical and experimental efforts are not very effective methods for understanding and developing treatment strategies^10^. Fortunately, mathematical modeling provides a cost-effective and highly efficient method of evaluating different strategies by describing quantitative relationships between many factors such as the number of cancer cells, the levels of drug toxicity, and the likelihood of drug resistance. Mathematical models also allow scientists to better study the impact of different factors such as tumor progression and drug injection rate on the performance of optimum drug protocols. Besides predicting drug delivery in solid tumors, the model presented in this study is capable of helping treatment scheduling. By predicting the FKCs after each treatment step and considering regrowth in the breaks, between treatments, the presented approach can be employed to compare treatment schedules with various parameters such as drug dose and treatment cycle frequency. The importance of choosing the optimal dosage as well as the drug holiday to achieve maximum drug benefit to the patient and treatment efficacy is shown in Figs. 2 and 3, demonstrating good qualitative compatibility with the literature^57–59^. Based on computational-experimental study of Howard et al.^57^, inter-treatment interval is a significant factor in determining the efficacy of successive dosing plans and identifying an optimal retreatment time. The effects of treatment scheduling—with varying treatment dose, duration, and length of drug holiday—on the drug resistance evolution in breast cancer have been investigated by Patwardhan et al.^59^.

Applying a method for predicting the effect of tissue transport on chemotherapeutic treatment scheduling can enhance efficacy. With the aim of predicting the impact of treatment schedules on drug efficacy, the computational approach proposed in this study integrates diffusion via tissue, cell-binding, and cellular internalization. As illustrated by simulations, efficacy is strongly dependent on tissue transport. The study proves that the most advantageous results are obtained after the administration of chemotherapy agents with intermediary bindings. Slow diffusivity drugs are all unsuccessful, and slow-clearance drugs are specifically beneficial. These results are in good agreement with the results of single-cycle treatment of Stylianopolous et al.^36^ and Moradi Kashkooli et al.^32^ based on FKCs for drug DOX.

There exist two major approaches for treating tumors through chemotherapy: (i) using successive treatment cycles, (ii) reducing the tumor to a prespecified size via chemotherapy and then applying an adjuvant therapy. As the results show, at the initial treatment cycles, when the tumor size is larger and as a result the number of TCs is higher, treatment is much more effective in decreasing the number of TCs. Thus, in applying chemotherapy, the smaller the tumor size, the higher the treatment cycles number, and the greater the side effects. Consequently, the best available solution is to first decrease the tumor to a prespecified size through chemotherapy, and then eradicate the last of the tumor using an adjunct therapy.

It is worth mentioning that the results of present study rely on an image extracted from the literature to drive the calculations; so, by evaluating only one sample vascular structure, possible dependency of the results on the chosen image should be considered. Additionally, the structure of capillary network changes during tumor growth. Based on the study of Zhuang et al.^60^, the role of angiogenesis in tumor growth could potentially affect the fluid flow modeling accuracy. This issue is not considered in this study due to the lack of enough images from different stages of tumor growth as well as the fact that time-dependent variations in microvessels cannot be applied to such a microvasculature geometry extracted from a real image, but it will be considered in our future works.

Results of the case study show that the survival rates of TCs after several treatments offer significant prognostic insight about the survival rate and the cell regrowth percentage. Being able to evaluate the efficacy of a treatment scenario is very helpful in clinical situations. Results on toxicity and efficacy indicate that our model is highly complementary to PK/PD models and it thus provides a promising pathway for designing effective protocols in oncology. Taking into account the above-mentioned modules, simulations with the new model would offer insights into important drug delivery mechanisms within tumors and simultaneously provide a better and more feasible appraisal of tumor therapeutics. In other words, our computational tool and its results can provide guidelines for more-accurate assessment of treatment plans and enhance the results of preclinical in vivo tests and early clinical trials. It can also be used for different chemotherapy drugs as well as various treatment methods such as nanomedicine and hyperthermia.

## Material and methods

Empirical and mathematical methods have been attempted to explain the basics of growth and kinetics of TCs as well as chemotherapy treatment. These systems offer better understanding of tumor-growth characteristics, tumor shrinkage, the key principles of chemotherapy treatment, and ultimately, drug doses and scheduling. In Supplementary File, a comprehensive study is conducted on basic tumor growth models, current methods of treatment modeling, drawbacks of existing treatment models, and treatment efficacy calculations. Therefore, a new method is proposed that resolves the weaknesses of previous models. It is necessary to mention that the present study specifically focuses on the models that can be applied to clinical data. Details about the case study and computational domain, parameter values, numerical simulation setting, and boundary conditions are discussed in Supplementary File.

### Novel proposed approach

After a comprehensive review of the related literature, we have concluded that there have been many studies on the modeling of fluid flow and drug delivery in tumors, tumor growth, and chemotherapy treatment in solid tumors, but each of these studies explores only one or rarely two of these concepts. Moreover, there are only very limited connections between these concepts. A simultaneous examination of all these effects had not been carried out until the present study. Among its valuable contributions, it applies a mathematical approach to solve the various problems in the field of drug delivery and treatment, modifies and further develops previous models, examines the effects of the proposed model on treatment, and calculates treatment efficacy. In this study, we use FKCs to evaluate dug delivery and treatment efficacy, and then propose a comprehensive model that includes interstitial fluid flow, drug delivery, tumor growth, and treatment of solid tumors. Accordingly, the proposed approach has the following characteristics:

- Fluid flow modeling of the interstitium and capillary network, drug delivery (spatiotemporal distribution of drug concentration) in a tumor with a real/synthetic geometry, and also specific attention to the details of the problem (considering issues such as drug binding through its ligands to cancer-cell receptors, cellular uptake, natural drug decay, to name a few);
- A mathematical model and numerical simulation of drug delivery used for predicting the effect of treatment, assuming tumor growth in the intervals between two consecutive treatments. However, the role of angiogenesis in tumor growth could potentially impact the fluid flow modeling accuracy. Therefore, if the dynamic nature of vascular structure inside the solid tumor could be included, the accuracy of the model could be potentially strengthened;
- Investigation of the effectiveness of each phase of treatment by computing the FKCs, the area under curve (AUC) for concentration, and the fraction of surviving cancer cells (FSCs);
- Proposal of an integrated and comprehensive model for evaluating solid tumor treatment, considering fluid flow, drug delivery, and treatment, simultaneously.

**Figure 5:**
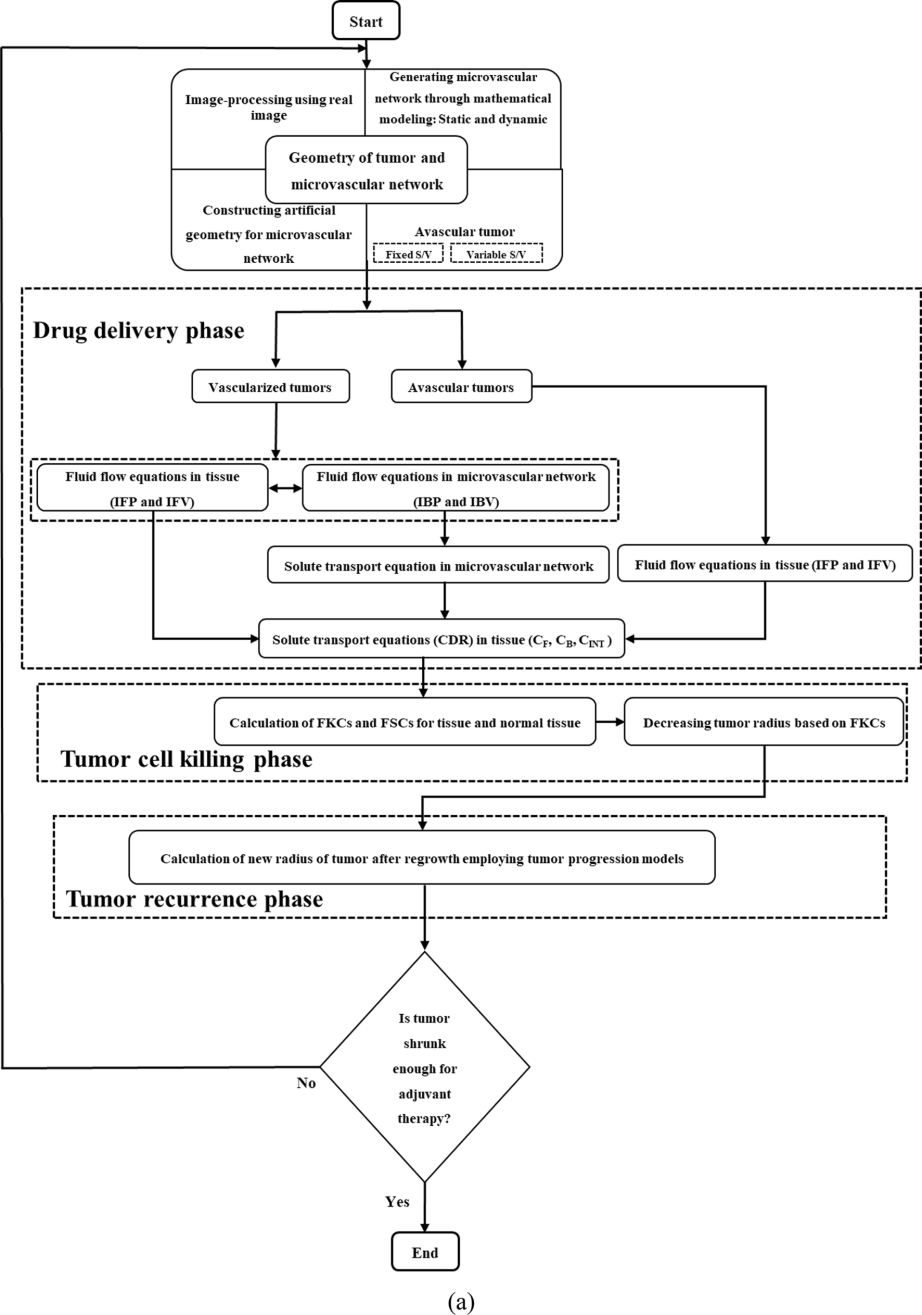

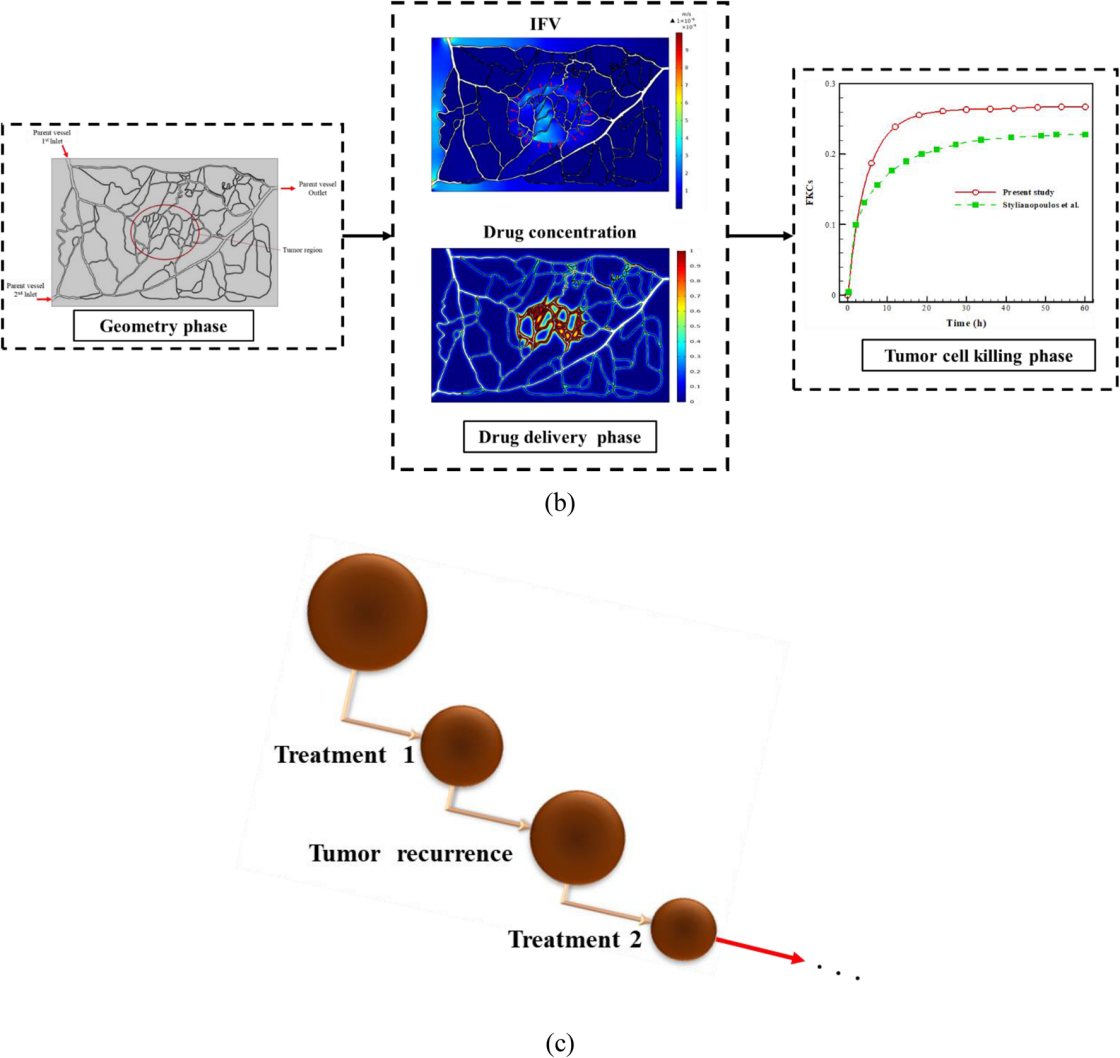
Integrated and comprehensive computational approach presented for evaluating solid tumor treatment. (**a**) Flowchart of novel approach proposed for assessing efficacy of sequential treatment cycles; (**b**) Different phases of modeling for a single treatment cycle on the present case study for drug DOX, including geometry generation phase, drug delivery phase, and tumor cell killing phase. It is worth-mentioning that the right-hand side figure demonstrates a comparison of the results of this study with Stylianopolous et al.^36^; (**c**) A schematic of proposed approach based on sequential treatment cycles and tumor recurrence between two consecutive treatments for evaluating different treatment schedules.

Different phases of modeling solid tumor treatment with chemotherapy drugs are illustrated in Fig. 5. After creating geometry, this flowchart completely summarizes the procedures and characterizes those repeated in several treatment phases: the (i) drug delivery phase; (ii) tumor-cell killing phase; and (iii) tumor recurrence phase.

### Mathematical modeling

The governing equations for the case study cover fluid flow in the microvasculature and interstitium, drug transport, efficacy of chemotherapy, and eventually, tumor cell survival.

### Intravascular fluid flow

Poiseuille law is employed to acquire the intravascular pressure and velocity in the capillaries Here, the Poiseuille law is corrected due to blood physics in the capillary network, which is severely affected by hematocrit and blood viscosity. Thus, the apparent viscosity is employed as a substitute for the viscosity term, as below^61^:

1 $$\mu_{app} = \mu_{plasma} \cdot \mu_{rel}$$

where $\mu_{app}$, $\mu_{plasma}$, and $\mu_{rel}$ represent the apparent, plasma, and relative viscosities, respectively. $\mu_{rel}$ is calculated through the following equations^61^:

2 $$\mu_{rel} = \left[ {1 + \left( {\mu_{45} - 1} \right)\frac{{\left( {1 - H} \right)^{\xi } - 1}}{{\left( {1 - 0.45} \right)^{\xi } - 1}}\left( {\frac{D}{D - 1.1}} \right)^{2} } \right]\left( {\frac{D}{D - 1.1}} \right)^{2}$$

3 $$\mu_{45} = \left( {6 e^{ - 0.085D} } \right) + 3.2 - \left( {2.44 e^{{ - 0.06D^{0.645} }} } \right)$$

4 $$\xi = \left( {0.8 + e^{-0.075D} } \right)\left( { - 1 + \frac{1}{{1 + 10^{ - 11} D^{12} }}} \right) + \frac{1}{{1 + 10^{ - 11} D^{12} }}$$

where $\mu_{45}$ is the blood’s relative apparent viscosity for the fixed hematocrit of 0.45, *D* is the microvessels’ diameter, and ξ is the dependency of viscosity to the hematocrit.

The fluid exchange rate from the capillaries into the tissue is obtained by Starling's law^32^:

5 $$Q_{t} = \pi DLL_{p} \left( {P_{b} - P_{i} - \sigma_{s} \left( {\pi_{b} - \pi_{i} } \right)} \right)$$

in which $Q_{t}$ is the transvascular rate, *L*_*p*_ is the hydraulic conductivity, *L* is the vessel wall length, *P*_*i*_ is IFP, and *P*_*b*_ is intravascular pressure. *π*_*b*_ and *π*_*i*_ respectively represent the osmotic pressure for capillaries and interstitial fluid, and *σ*_*s*_ demonstrates mean of the osmotic reflection coefficient for proteins of plasma.

### Interstitial fluid flow in tissue

The momentum and continuity equations in biological tissue as porous medium, assuming the existence of the source and sink terms, are as follows^32,33^:

6 $$v_{i} = - \kappa \nabla P_{i}$$

7 $$\nabla \cdot \upsilon_{i} = \phi_{B} - \phi_{L}$$

where *v*_*i*_ is interstitial fluid velocity, *P*_*i*_ is IFP, $\kappa$ is the tissue hydraulic conductivity, $\phi_{B}$ is the exchange rate of fluid flow from the capillaries to the interstitium, and $\phi_{L}$ is the exchange rate from interstitium to the lymph system. These two terms are obtained by the following equations^32,33^:

8 $$\phi_{B} = L_{P} \left( \frac{S}{V} \right)\left( {P_{b} - P_{i} - \sigma_{s} \left( {\pi_{B} - \pi_{i} } \right)} \right)$$

9 $$\phi_{L} = L_{PL} \left( \frac{S}{V} \right)_{L} \left( {P_{i} - P_{L} } \right)$$

where *S*/*V* represents the surface area per volume of the microvessels, $L_{PL} \left( \frac{S}{V} \right)_{L}$ is the lymph filtration coefficient, and *P*_*L*_ is the hydrostatic pressure of lymph.

### Drug transport in tissue

The Convection–Diffusion-Reaction (CDR) set of equations, according to Fick’s law, are employed for transport of drug as^62^:

10 $$\frac{{\partial C_{F} }}{\partial t} = - \nabla \cdot \left( {v_{i} C_{F} } \right) + \nabla \cdot \left( {D \nabla C_{F} } \right) - \frac{1}{\varphi }K_{ON} C_{rec} C_{F} + K_{OFF} C_{B} + \left( {\Phi_{B} - \Phi_{L} } \right)$$

11 $$\frac{{\partial C_{B} }}{\partial t} = \frac{1}{\varphi }K_{ON} C_{rec} C_{F} - K_{OFF} C_{B} - K_{INT} C_{B}$$

12 $$\frac{{\partial C_{INT} }}{\partial t} = K_{INT} C_{B}$$

where *C*_*F*_, *C*_*B*_, *C*_*INT*_, *C*_*rec*_ are the free drug concentration, bound drug, internalized drug, and drug concentration at the cell-surface receptors, *v*_*i*_ is IFV, D is the diffusion coefficient, and *φ* is the tumor volume fraction accessible to drugs. *K*_*ON*_, *K*_*OFF*_, and *K*_*INT*_ are the association, dissociation, and cellular internalization rate constants. $\Phi_{{\text{B}}}$ is the drug transport rate from microvessels to the tissue, and $\Phi_{{\text{L}}}$ is the drug transport rate from the tissue to the lymph system. These two terms are given as^62^:

13 $$\Phi_{B} = \phi_{B} \left( {1 - \sigma_{f} } \right)C_{P} + \frac{PS}{V}\left( {C_{P} - C_{F} } \right)\frac{Pe}{{e^{Pe} - 1}}$$

14 $$Pe = \frac{{\phi_{B} \left( {1 - \sigma_{f} } \right)V}}{PS}$$

15 $$\Phi_{L} = \phi_{L} C_{F}$$

in which *Pe* is the Péclet number, σ_*f*_ is the filtration reflection coefficient, *P* is the permeability of microvasculature, and *C*_*p*_ is the concentration of drug in plasma ($C_{P} = C_{0} \exp ( - t/K_{d} )$).

### Efficacy of chemotherapy drug

Despite its cardiotoxicity, DOX is frequently employed in tumor treatment. DOX is a standard-of-care, DNA-damaging agent employed in the treatment of multiple tumors. Using the internalized drug concentration (*C*_*INT*_), we calculate the efficacy of DOX (i.e., FKCs) and also its side effects on normal tissue as follows^32^:

16 $$FKCs = 1 - S_{F} = 1 - \exp \left( { - \omega \cdot C_{INT} } \right)$$

where *ω* is a constant determined for DOX in the literature^63,64^ and *S*_*F*_ represents the FSCs after delivery of the drug.

### Tumor cell survival

The number of TCs after a period of time is equal to the intervals between chemotherapy sessions (*n*_*i*_), which is obtained through Gompertz’s model^65^. This equation is a function of three parameters: the number of TCs surviving after the *i*th treatment (*N*_*i*_), the number of saturated cells after a very long period ($N_{\infty }$), and eventually the rate of tumor progression (*b*).

17 $$n_{i} (t) = N_{i} \exp \left\{ {Ln \left( {\frac{{N_{\infty } }}{{N_{i} }}} \right)\left[ {1 - \exp ( - bt)} \right]} \right\}$$

The number of surviving TCs as a consequence of the difference between the cells number after (*N*_*i*_) and before (*N*_*i-1*_) each treatment will also be examined as a standard for evaluating efficacy of treatment. In this model, *S*_*F*_ is also defined, as^37^:

18 $$(S_{F} )_{i} = \frac{{N_{i} }}{{N_{i - 1} }}$$

The primary numbers of TCs here, adopted from the literature^65^, are N_0_ = 5 × 10^9^, N_∞_ = 3.1 × 10^12^, and b = 0.0283 month^−1^. The number of cells for 1-cm thick healthy tissue was considered to be N_1_ = 4.64 × 10^12^^65^. The number of surviving cells is assumed to depend on volume of tumor; i.e., as the number of cells reduces, the tumor shrinks and radius of tumor reduces. The ratio of the density of healthy tissue to the density of tumor cell is considered to be 0.2^66^, and it is also assumed that the structure of capillary network in the computational field does not change after each treatment cycle. In addition, the regrowth of healthy tissue cells was examined by using Eq. (17) with the assumption that the growth rate of healthy tissue is half that of the tumor^37^.

### Simulation cases

First, the effect of three different approaches to chemotherapy administration—MTD^67^, LDC, also called MC^16^, and CS^22^, as a kind of adaptive therapy—are examined based on treatment evaluation. Then, the impacts of two effective parameters in treatment scheduling (i.e., administration frequency and dose) are investigated and discussed in detail. In the following, a full parametric study is carried out to examine the effects of drug characteristics—diffusion, binding affinity, and half-life—on treatment efficacy. It should be mentioned that all partial differential equations (PDEs) are coupled and solved using finite element based COMSOL Multiphysics (version 5.5a) commercial software.

### Verification of numerical results

In the present study, verification is carried out considering the same equations as the literature^36^ to obtain the variations of FKCs parameter over time (Fig. 5b). As is clear, there is a good agreement between the results of this study and the results of the previously-published study so that by taking into account the actual geometry and physics, the FKCs parameter has a similar variation pattern. However, assuming the same circumstances, its amount has almost 4% difference, because of the differences in geometry of tumor, computational field, and structure of microvascular network. The maximum value of intravascular pressure in this study is 3330 Pa compared to 3520 Pa obtained by Shojaee et al.^68^, demonstrating a 5.4% difference. The maximum intravascular velocity value is 0.16 m/s in the current study, which is in good agreement with Moradi Kashkooli et al.^32^ and Shojaee et al.^68^. The obtained value for IFP is consistent with the experimental investigations of Huber et al.^69^ and Arifin et al.^70^, and the numerical studies of Sefidgar et al.^27^, Soltani et al.^71^, and Moradi Kashkooli et al.^32,72^. The IFV amount (median value ~ 0.031 µm/s) is also in good correspondence with numerical studies of Pishko et al.^73^, Souri et al.^74^, and Zhao et al.^75^; and in the same order with the value reported by experimental study of Homopland et al.^76^.

## Supplementary Information

Supplementary Information.

## Data Availability

All data used for this study are available from the author upon request.
