# Supplementary material for: Evaluation of solid tumor response to sequential treatment cycles via a new computational hybrid approach
Source: Sci Rep. 2021 Nov 2;11:21475. doi: 10.1038/s41598-021-00989-x (PMC8563754; doi:10.1038/s41598-021-00989-x)
Supplement: Supplementary file 1 — Supplementary Information. [file 41598_2021_989_MOESM1_ESM.docx]

**Supplementary File**

**Evaluation of Solid Tumor Response to Sequential Treatment Cycles via a**

**New Computational Hybrid Approach**

Farshad Moradi Kashkooli, M. Soltani

**S.1. Basic tumor growth models**

Most studies employ ordinary differential equations (ODEs) to characterize tumor burden variation. Natural tumor growth models with no treatments typically include many basic functions, as summarized in Table S1. In this table, *a* and *b* is constants, *k*_d_ is shrinkage rate constant resulting from drug treatment, *k*_g_ is growth rate constant, *T* is tumor burden, *T_max_* is carrying capacity, λ_0_ is exponential growth rate, and λ_1_ is linear growth rate.

| **Table S1.** Basic functions of tumor growth | | | | |
| --- | --- | --- | --- | --- |
|  | **Tumor growth model** | **Equations** | **Eq. number** | **References** |
| 1 | Linear | $\frac{dT}{dt}=k_{g}$ | (1) | [1] |
|  |  | $\frac{dT}{dt}=k_{g}-k_{d}\cdot T$ | (2) | [2] |
| 2 | Exponential | $\frac{dT}{dt}=k_{g}\cdot T$ | (3) | [3] |
|  |  | $\frac{dT}{dt}=k_{g}\cdot T-k_{d}\cdot T$ | (4) | [4] |
| 3 | Logistic | $\frac{dT}{dt}=k_{g}\cdot T\cdot(1-\frac{T}{T_{max}})$ | (5) | [5] |
| 4 | Gompertzian | $\frac{dT}{dt}=k_{g}\cdot T\cdot ln(\frac{T}{T_{max}})$ | (6) | [6] |
|  |  | $\frac{dlnT}{dt}=a-blnT$ | (7) | [7] |
| 5 | Combination of exponential and linear | $\frac{dT}{dt}=\frac{\lambda_{0}T}{{[1+{(\frac{\lambda_{0}}{\lambda_{1}}\cdot T)}^{20}]}^{\frac{1}{20}}}$ | (8) | [8] |

The linear growth of a tumor has a constant zero-order growth rate (Eq. 1) [9]. This model, for instance, has been used to describe natural tumor growth in metastatic renal cell carcinoma. Exponential growth of a tumor considers that the progression rate depends on the tumor burden (Eq. 3). A popular tumor growth inhibition (TGI) model has been proposed by Claret *et al.* to describe tumor progression’s nature [3,9].

Linear and exponential tumor progression models were developed by introducing a first-order shrinkage term presenting natural death of the tumor. For instance, a model using linear growth and first-order shrinkage (Eq. 2) was utilized for describing the tumor’s natural growth in patients who had advanced solid lesions [2]. Exponential growth with first-order shrinkage (Eq. 4) was also applied in part of a model structure to characterize the natural progression of pediatric neuroblastoma on the basis of tumor volume computations [4]. A similar model structure was also used to describe the variations in prostate cancer burden presented by prostate-specific antigen (PSA) levels [10].

The logistic and Gompertz models reflect a biologically rational variation in the rate of growth with increasing tumor burden, since they compare the unlimited growing approaches of these progression models [11]. The logistic progression model supposes that tumor progression is restricted by carrying capacity (Eq. 5) [9], while the Gompertz model considers the tumor progression rate to reduce with time (Eqs. 6 and 7) [9,12]. A great number of clinical investigations [5,6,12] along with simulation studies have utilized logistic and Gompertz models [7,13].

Additionally, a hybrid of exponential and linear models (Eq. 8) has been proposed to describe tumor progression in patients, even though it was initially introduced to characterize xenograft tumor dynamics [14]. This hybrid model structure considers exponential growth (first order) to shift to linear growth (zero order) upon meeting a threshold. It has been widely employed for describing the natural tumor growth in patients with neurofibromatosis type 2 [8].

In general, the exponential growth model has been the most common choice in clinical research on the natural tumor progression functions (Eqs. 1-8) as a permanent part of the tumor dynamics models. The logistic progression model was usually desirably in cases when the maximum capacity of a tumor was constant. Selecting the basic functions may also be dependent on a model’s fit to the data. Multiple accessible pre-treatment tumor size measurements could help obtain the most efficient fit for a natural progression model and allow a more precise prediction of the tumor’s natural progression rate.

**S.2. Empirical methods for cancer treatment**

Various methods have been proposed for treatment modeling with chemotherapy, each originating from a different medical approach. In other words, tumor shrinkage in response to treatment is commonly quantified through empirical drug-induced shrinkage equations. The most important of these approaches adopted from the literature are presented in brief in Table S2. In this table, *D* demonstrates damaged cells, *E_max_* represents maximal fraction of inhibition*,* IC_50_ is the drug dose that produces 50% of *E_max_*, *k*_d_ shows shrinkage rate constant of tumor resulting from drug treatment, *k*_g_ indicates growth rate constant, *k*_g_ʹ illustrates tumor growth rate constant under treatment, *T* is tumor burden, and λ represents constant of decay rate of treatment efficacy. *f*(*S*) and *f*(*T*) are growth function of sensitive cells (*S*) and tumor tissue (*T*), respectively;

| **Table S2.** Empirical model structures describing therapeutic effect | | | | |
| --- | --- | --- | --- | --- |
|  | **Treatment model** | **Equations** | **Eq. number** | **References** |
| 1 | First-order (“log-kill” pattern) | $\frac{dT}{dt}=f(T)-k_{d}\cdot T$ | (9) | [1] |
| 2 | Dose-dependent | $\frac{dT}{dt}=f(T)-k_{d}\cdot\mathrm{Dose}\cdot T$ | (10) | [4,5] |
| 3 | Dose-dependent with resistance (TGI model) | $\frac{dT}{dt}=f(T)-k_{d}\cdot e^{-\lambda t}\cdot Dose\cdot T$ | (11) | [3] |
| 4 | Nonlinear drug-dose | $k_{g}^{'}=k_{g}\cdot T\cdot(1-\frac{E_{max}\cdot\mathrm{Dose}}{\mathrm{IC}_{50}+\mathrm{Dose}})$ | (12) | [2] |

A log-kill approach is normally incorporated for predicting the effects of treatment, whereby the tumor shrinkage rate caused by drug treatment is assumed to be proportional to the tumor burden [11]. Employing Eq. 9 is the simplest way of using this pattern, which has been adopted to describe the results of treating metastatic renal cell carcinoma patients with the drug Everolimus [1].

It may also be considered that the drug-induced shrinkage rate is dependent on the drug dose, i.e., the area under the curve (AUC) of concentration during time or drug dosage. Eq. 10 can help to calculate a linear dose-effect relationship [4,5]. In the meantime, drug resistance may be considered by adding an *e*^-^*^λ^*^·^*^t^* term based on Eq. 11 so as to calculate the gradual drop of drug effect over time (Eq. 12).

In addition to the linear dose-effect relationship, a non-linear relationship can also be assumed to describe the effect of therapy specifically for targeted therapy [2]. An *E_max_* model is typically employed under this condition. Eq. 12 is an example adopted from a model in which the investigated drug is considered to impede the zero-order progression rate of an advanced solid tumor using the non-linear dose-effect relationship [2].

The characteristics of two significant empirical models characterizing treatment effect are demonstrated in Table S3.

| **Table S3.** Characteristics of two significant empirical models describing treatment impact [15,16] | | | |
| --- | --- | --- | --- |
|  | **Treatment model** | **Characteristics** | **Objective** |
| 1 | First-order (“log-kill”) | - Drug dependent - Parameters can differ for various dosage groups | - Specify therapeutic effect |
| 2 | Dose-dependent | - Longitudinal concentration data - Or PK model for simulating drug exposure or dose (as a criterion for drug dose) - Appropriate for various therapies and types of cancer - Appropriate for both mono and combo therapies | - Specify relationship between change of size of tumor and exposure on treatment - Treatment optimization |

In the mentioned models, treatment efficacy may be determined either by a drug-dependent approach or a dose-dependent one. When the research work is not concerned with examining the dose impact relationship, applying a model with drug-dependent tumor shrinkage is sufficient and information on drug doses is unnecessary. However, if a study aims to describe the relationships among drug exposure/dose and tumor response and/or treatment schedule optimization according to simulations, a dose-dependent treatment impact structure is needed. Predicting drug doses requires longitudinal concentration data for the development of PK model or a formerly published PK model. Furthermore, prior understanding of the mechanisms of treatment could also be necessary for determining treatment effects, particularly when models involving biological factors are utilized. For describing therapeutic impact, empirical techniques are more commonly applied to characterize the influence of different drug types in order to describe treatment effect, and so they are generally more applicable. Describing a tumor burden’s shrinkage rate induced by treatment is possible in proportion to drug dose or by applying parameters related to the drug, even though the second method does not allow a distinction between various schedules of dosing [8,17].

In addition, tumor recurrence can be taken into consideration through multiple approaches. Studies applying algebraic equations commonly describe a tumor’s shrinkage and regrowth using a single equation. With studies using ODEs, tumor recurrence is primarily described by dividing the tumor cells into two sections: one including drug-sensitive cells and the other one, drug-resistant cells.

**S.3. Drawbacks of existing treatment models**

In all of the studies mentioned, the focus has been only on tumor treatment, completely ignoring issues related to drug delivery processes and TME. In addition, all of these studies have been in the field of mathematical modeling using PK/PD models; no spatial-temporal modeling of drug delivery to solid tumors has been investigated with respect to their real geometry. Classic models of PK and PD, the former generally demonstrating the effect of the body on drugs, and the latter representing the effect of drugs on the body, both assume that drug concentration in the body has a uniform distribution. However, more-realistically, drug-accumulation kinetics in different regions of tumors can be analyzed using mass transport models that regard tissue as a porous media. Such models consider the mechanisms of mass transport and also drug delivery through the capillary network [18,19], and have two important features not found in the classic PK/PD models:

- The models focus on drug delivery and interstitial fluid flow processes and also heterogeneous drug distribution in tumors, whereas the PK/PD model assumes uniform distribution.
- The parameters of solute transport models have physiological and bio-chemical implications, and can represent the properties of tumor, drug, and drug delivery processes (e.g., intravascular pressure, microvascular density, diameter, permeability, and diffusion coefficient), whereas PK/PD method might involve a parameter to express all or some of the above parameters.

The mass transport model is a powerful tool for assessing the mechanisms limiting drug penetration into tumors, and it allows researchers to predict drug concentration within tumors. It is true that there are advantages to using a solute transport model instead of PK/PD models, but the lack of connection between drug delivery effects and treatment evaluation is problematic considering the tumor growth possible between consecutive treatments. To solve this issue, the fraction of killed cells (FKCs) and AUC have been introduced. On the other hand, many models that evaluate drug-efficacy use cell-death kinetics and systemic pharmacokinetics, but do not explicitly include transport in tissue level.

**S.4. Treatment efficacy calculations**

Following phagocytosis by tumor cells, drugs may damage the genes that cause cell division or interrupt the chemical process involved in cell division, both resulting in cell death. Drug cytotoxicity was initially assumed to be a function of the AUC, i.e., it is considered that the AUC can effectively predict treatment efficacy for some time [20]. Recently, treatment efficacy has been mainly obtained through calculating the FKCs, which are mainly dependent on the extracellular and intracellular drug concentrations, and various models have been presented to describe this relationship. Green et al. [21] and Eichholtz-Wirth [22] reported that the FKCs in tumors are a function of drug concentration in the extracellular matrix, while El-Kareh and Secomb [23] concluded that the FKCs depend mainly on drug concentration in the intracellular space. Despite these works, experimental results have demonstrated a non-linear relation between drug concentrations in extracellular and intracellular spaces [24]. The varying viewpoints on the FKCs parameter after treatment are summarized in Table 4. In this table, *K_sf_*, *T_sf_*, *C_d_*, *D_c_*, and *K_d_* represent survival fraction constant, half-life of Temozolomide (TMZ), drug concentration, pharmacodynamical parameter, and change of tumor cell density, respectively. *F* is the drug flux across the walls of blood vessel, *λ_k_* is the tumor cells’ death-rate, *V_T,0_* is the volume of tumor at the beginning of treatment (positive *f_kill_*), and *t* is time. In Eq. (20), *K_0_* and *K_1_* are modified Bessel functions of the second kind of orders 0 and 1, respectively. It should be noted that that *f_kill_* by IV drug injection is quantified as a function of a limited set of parameters, including the vascular density or blood volume fraction (BVF), the radial coordinate scaled with the drug diffusion penetration distance *L*, straight cylindrical blood vessel radius *r_b_*, and fraction of cells killed *in vitro*, which can all be directly measured from histopathology and monolayer cell culture tests.

| **Table S4.** Different formulae for obtaining FKCs for of treatment-efficacy evaluation | | | | |
| --- | --- | --- | --- | --- |
|  | **Study** | **Drug type** | **Equations** | **Eq. number** |
| 1 | Moradi Kashkooli *et al*. [18] | Doxorubicin (DOX) | $FKCs=1-\exp(-\omega\cdot C_{INT})$ | (13) |
|  | Stylianopolous *et al*. [25] |  |  |  |
|  | Kerr *et al*. [26] |  |  |  |
| 2 | Ebrahimi Zade *et al*. [27] | TMZ | $FKCs=1-\exp(-K_{sf}\cdot T_{sf}\cdot C_{d})$ | (14) |
| 3 | Chou *et al*. [28] | Each drug | $FKCs=1-\exp(-K_{s}\cdot AUC)=1-\exp(-K_{s}\cdot\int_{0}^{t} C_{i}(r,t)dt)$ | (15) |
|  | Zhang *et al*. [29] |  |  |  |
| 4 | Zhan *et al*. [24] | DOX | $\frac{dD_{c}}{dt}=-\frac{f_{\max}C_{i}}{EC_{50}+C_{i}}D_{c}+k_{p}D_{c}-k_{g}D_{c}^{2}$ | (16) |
| 5 | Ganz *et al.* [30] | Each drug | $\frac{dN}{dt}=-\alpha(\frac{L^{n}}{K_{d}+L^{n}})N$ | (17)  (18) |
|  |  |  | $FKCs(t)=1-\frac{1}{L}\int_{0}^{L} N(k,m,t,x)dx$ |  |
| 6 | Wang et al. [31]  Brocato et al. [32] | Each drug | $f_{kill}=\frac{F\cdot\lambda_{k}}{2V_{T,0}}t^{2}$ | (19) |
| 7 | Pascal et al. [33] | Each drug | $f_{kill}=f_{kill}^{M}\left( \sigma_{0} \right)\cdot BVF\cdot\frac{2\sqrt{BVF}\cdot K_{1}\left( \frac{r_{b}}{L} \right)-2K_{1}(\left( \frac{r_{b}}{L} \right)/\sqrt{BVF})}{\sqrt{BVF}\cdot\left( \frac{r_{b}}{L} \right)\cdot\sqrt{BVF}\cdot K_{b}\left( \frac{r_{b}}{L} \right)\cdot(1-BVF)}$ | (20) |

**S.5. Case study**

In order to assess the proposed model’s performance for chemotherapy, a case study is performed on the geometry obtained from an image of a solid tumor. In the following, this case study is carefully described, and the results are discussed in detail.

**S.5.1. Computational domain**

A two-dimensional computational domain in the presence of a tumor (with geometry obtained from a real mouse tumor) surrounded by normal tissue will be explored in this study. The geometry of the tumor and the capillary network have been obtained through image processing. The original image is taken from Roudnicky et al. [34] and then, using image-processing techniques, the images are converted to a software-usable input for further analysis. The two-dimensional computational domain illustrated in Fig. S1 will be incorporated in the study’s various phases.

| 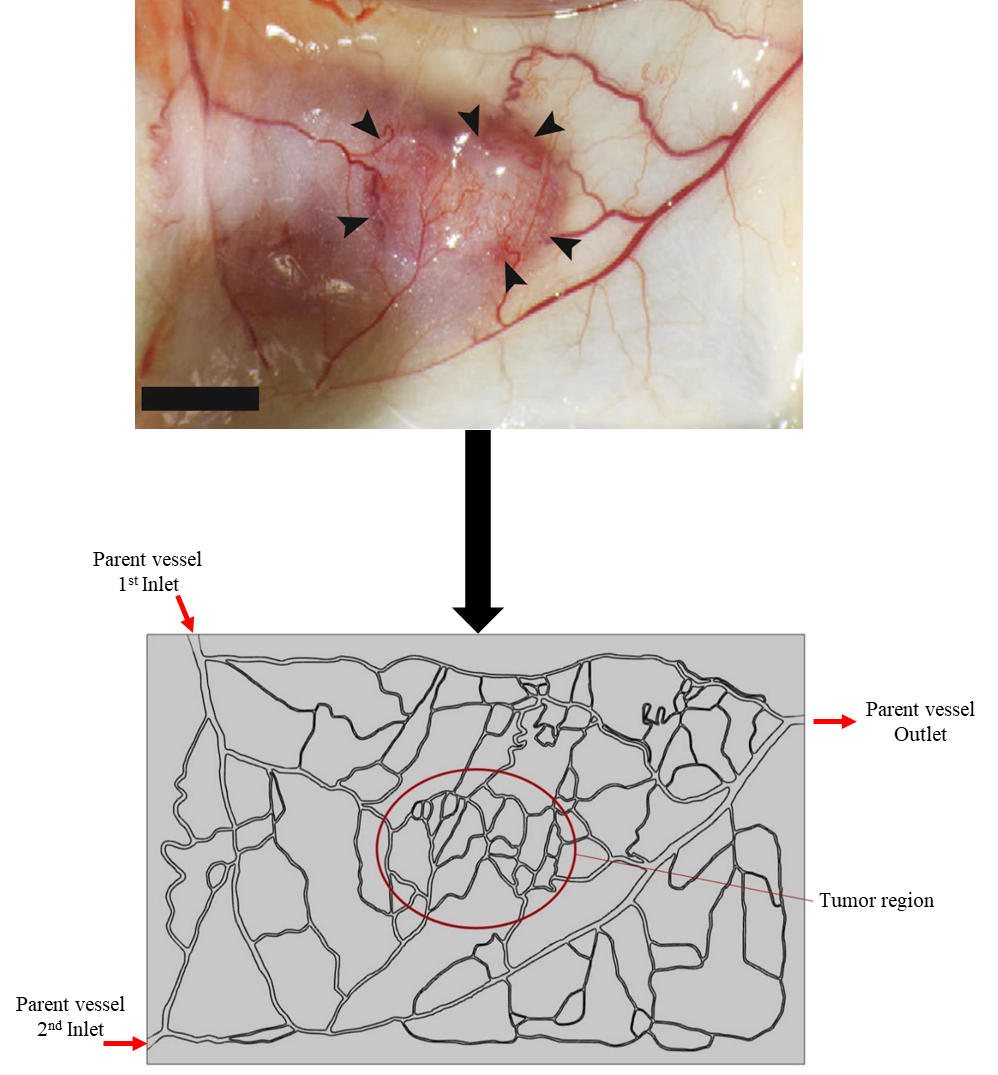 |
| --- |
| (a) Initial image; adopted with permission from [34], all rights reserved. |
| **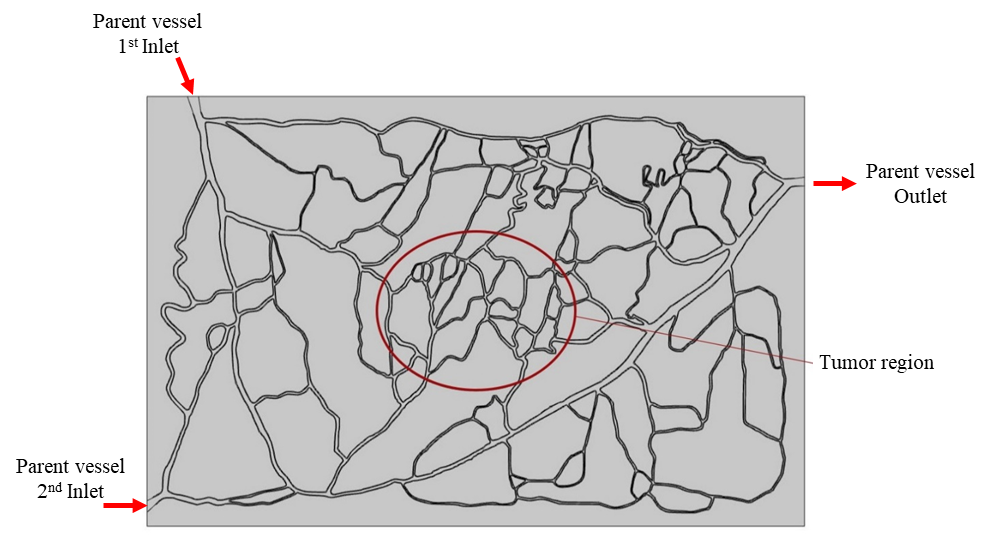** |
| (b) Generated computational domain along with inlet and outlet parent vessels. |
| **Fig. S1.** Computational domain considered in this study |

**S.5.2. Parameter values**

The drug properties in normal and tumor tissues are provided in Table S5. Although the numerical method used is applicable to any type of drug, the properties of DOX are used for this case study. The baseline values of interstitial transport properties for normal and tumor tissues are also presented, in Table S5.

| **Table S5.** Values of the parameters used in the modeling of fluid flow and DOX delivery | | | | | | | |
| --- | --- | --- | --- | --- | --- | --- | --- |
| **Reference** | **Value** | | **Type of tissue** | | **Unit** | **Parameter** | |
| Parameters for drug delivery modeling | | | | | | | |
| [35] | 1.58×10^-10^ | | Healthy tissue | | m^2^/s | D | |
|  | 3.4×10^-10^ | | Tumor | |  |  |  |
| [35] | 3.57×10^-7^ | | Healthy tissue | | m/s | *P* | |
|  | 3×10^-6^ | | Tumor | |  |  |  |
| [18] | 0.35 | | Both | | *-* | *σ_f_* | |
| [18] | 1.50×10^4^ | | Both | | 1/(M·s) | K_ON_ | |
| [18] | 8×10^-3^ | | Both | | 1/s | K_OFF_ | |
| [18] | 5×10^-5^ | | Both | | 1/s | K_INT_ | |
| [25] | 0.4 | | Both | | *-* | *φ* | |
| [25] | 1×10^-5^ | | Both | | M | C_rec_ | |
| [36] | 0.6603 | | Both | | m^3^/mole | *ω* | |
| Parameters for interstitial fluid flow modeling | | | | | | | |
| [37] | | 20 | Healthy tissue | mmHg | | | π_b_ |
|  |  | 20 | Tumor |  |  |  |  |
| [37] | | 10 | Healthy tissue | mmHg | | | π_i_ |
|  |  | 15 | Tumor |  |  |  |  |
| [37] | | 0.91 | Healthy tissue | *-* | | | *σ_s_* |
|  |  | 0.82 | Tumor |  |  |  |  |
| [37 | | 0.36×10 | Healthy tissue | cm/((mmHg)$\cdot$s) | | | *L_p_* |
|  |  | 2.8×10^-7^ | Tumor |  |  |  |  |
| [37] | | 8.53×10^9^ | Healthy tissue | cm^2^/((mmHg)$\cdot$s) | | | $\kappa$ |
|  |  | 4.13×10^-9^ | Tumor |  |  |  |  |
| [38] | | 0.0 | Both | Pa | | | P_L_ |

**S.5.3. Numerical simulation and boundary conditions**

The flowchart and coupling of various steps of the proposed approach for a drug delivery system are demonstrated in Fig. 1 in the main file. All partial differential equations (PDEs) are coupled and solved using finite element based Comsol Multiphysics (version 5.5a) commercial software. Linear discretization method is employed for discretizing the fluid flow and solute transport equations. To capture the heterogeneity of the capillary network’s geometry, triangular elements are used for grid generation. For testing the mesh independency, three different meshes are created, and among them, fine-type mesh is chosen because of its accurate results and lower computational costs. The simulations are carried out on a system using an Intel 7^th^-generation processor with 2.8 GHz CPU and a 16 GB memory.

Two boundaries are considered in assessing the boundary conditions: the one between the tumor and the normal tissue (i.e., the internal boundary), and the one at the outer edge of the computational domain (i.e., the external boundary). The boundary conditions of this study are set out in Table S6. The initial value for IFP and the concentration is assumed to be zero. The inlet and outlet pressures of the parent vessels are chosen according to the real physiological circumstances expressed in [39] so the pressure in both inlets is considered to be 25 mmHg and the pressure in the outlet is assumed to be 10 mmHg.

| **Table S6.** Boundary conditions for the presented case study | | |
| --- | --- | --- |
| Zone | Boundary conditions | |
|  | Interstitial fluid flow | Drug transport |
| Internal boundary | $-\kappa_{t}\nabla P_{i}\left\lfloor\Omega^{-}=-\kappa_{n}\nabla P_{i}\left\lfloor\Omega^{+} \right. \right.$  $P_{i}\left\lfloor\Omega^{-}=P_{i}\left\lfloor\Omega^{+} \right. \right.$ | $(-D_{eff}^{t}\nabla C+\upsilon_{i}C) \left\lfloor\Omega^{-}=({-D}_{eff}^{n}\nabla C+\upsilon_{i}C) \right.\left\lfloor\Omega^{+} \right.$  $C_{i}\left\lfloor\Omega^{-}=C_{i}\left\lfloor\Omega^{+} \right. \right.$ |
| External boundary | $P_{i}=\mathrm{Constant}$ | $-n\cdot\nabla C=0$ |

**References**

[1] Stein, A. *et al*. Dynamic tumor modeling of the dose–response relationship for everolimus in metastatic renal cell carcinoma using data from the phase 3 RECORD-1 trial. BMC Cancer **12**, 311 (2012).

[2] De Buck, S.S. *et al*. Population pharmacokinetics and pharmacodynamics of BYL719, a phosphoinositide 3-kinase antagonist, in adult patients with advanced solid malignancies. *Br. J. Clin. Pharmacol.* 78, 543–555 (2014).

[3] Claret, L. *et al*. Model-based prediction of phase III overall survival in colorectal cancer on the basis of phase II tumor dynamics*. J. Clin. Oncol*. **27**, 4103–4108 (2009).

[4] Panetta, J.C., Schaiquevich, P., Santana, V.M. & Stewart, C.F. Using pharmacokinetic and pharmacodynamic modeling and simulation to evaluate importance of schedule in topotecan therapy for pediatric neuroblastoma. *Clin. Cancer Res.* **14**, 318–325 (2008).

[5] Ribba, B. *et al*. A tumor growth inhibition model for low-grade glioma treated with chemotherapy or radiotherapy. *Clin. Cancer Res.* **18**, 5071–5080 (2012).

[6] Belfatto, A. *et al*. Adaptive mathematical model of tumor response to radiotherapy based on CBCT data. *IEEE J. Biomed. Health Inform.* **20**, 802–809 (2016).

[7] Yu, R.X. & Holmgren, E. Endpoints for agents that slow tumor growth. *Contemp. Clin. Trials* **28**, 18–24 (2007).

[8] Ouerdani, A., Goutagny, S., Kalamarides, M., Troconiz, I.F. & Ribba, B. Mechanismbased modeling of the clinical effects of bevacizumab and everolimus on vestibular schwannomas of patients with neurofibromatosis type 2. *Cancer Chemother. Pharmacol.* **77**, 1263–1273 (2016).

[9] Ribba, B. *et al*. A review of mixed-effects models of tumor growth and effects of anticancer drug treatment used in population analysis. *CPT Pharmacometrics Syst. Pharmacol.* 3, e113 (2014).

[10] Hirata, Y., Azuma, S. & Aihara, K. Model predictive control for optimally scheduling intermittent androgen suppression of prostate cancer. *Methods* **67**, 278–281 (2014).

[11] Barbolosi, D., Ciccolini, J., Lacarelle, B., Barlesi, F. & Andre, N. Computational oncology–mathematical modelling of drug regimens for precision medicine. *Nat. Rev. Clin. Oncol.* **13**, 242–254 (2016).

[12] Bender, B.C., Schindler, E. & Friberg, L.E. Population pharmacokinetic-pharmacodynamic modelling in oncology: a tool for predicting clinical response. *Br. J. Clin. Pharmacol.* **79**, 56–71 (2015).

[13] Bethge, A., Schumacher, U. & Wedemann, G. Simulation of metastatic progression using a computer model including chemotherapy and radiation therapy. *J. Biomed. Inform.* **57**, 74–87 (2015).

[14] Simeoni, M. *et al*. Predictive pharmacokinetic-pharmacodynamic modeling of tumor growth kinetics in xenograft models after administration of anticancer agents. *Cancer Res.* **64**, 1094–1101 (2004).

[15] Benzekry S., Pasquier E., Barbolosi D., Lacarelle Bruno, Barlési F., Nicolas André, Joseph Ciccolini, Metronomic reloaded: Theoretical models bringing chemotherapy into the era of precision medicine. *Seminars in Cancer Biology* **35**, 53–61 (2015).

[16] Yin, A., Moes, D.J.A.R., van Hasselt, J.G.C., Swen, J.J., & Guchelaar, H.J. A Review of Mathematical Models for Tumor Dynamics and Treatment Resistance Evolution of Solid Tumors. *CPT Pharmacometrics Syst Pharmacol.* **8**(10), 720–737 (2019).

[17] Hansson, E.K. *et al*. PKPD Modeling of VEGF, sVEGFR-2, sVEGFR-3, and sKIT as predictors of tumor dynamics and overall survival following sunitinib treatment in GIST. *CPT Pharmacometrics Syst. Pharmacol.* **2**, e84 (2013).

[18] Moradi Kashkooli, F., Soltani, M., & Hamedi, M.H. Drug delivery to solid tumors with heterogeneous microvascular networks: Novel insights from image-based numerical modeling. *European Journal of Pharmaceutical Sciences*, **151**, 10539, (2020).

[19] Sefidgar, M., Soltani, M., Raahemifar, K., Sadeghi, M., Bazmara, H., Bazargan, M., & Mousavi Naeenian, M. Numerical modeling of drug delivery in a dynamic solid tumor microvasculature. *Microvascular Research*. **99**, 43–56 (2015).

[20] Nagai, N. & Ogata, H. Quantitative relationship between pharmacokinetics of unchanged cisplatin and nephrotoxicity in rats: Importance of area under the concentration-time curve (AUC) as the major toxicodynamic determinant in vivo. *Cancer Chemoth. Pharmacol.* **40**, 11–18 (1997).

[21] Greene, R.F., Collins, J.M., Jenkins, J.F., Speyer, J.L., & Myers, C.E. Plasma pharmacokinetics of adriamycin and adriamycinol: implications for the design of in vitro experiments and treatment protocols. *Cancer Research*. **43**(7), 3417–3421 (1983).

[22] Eichholtz-Wirth, H. Dependence of the cytostatic effect of adriamycin on drug concentration and exposure time in vitro*. Br. J. Cancer* **41**(6), 886–891 (1980).

[23] El-Kareh, A.W. & Secomb, T.W. A mathematical model for comparison of bolus injection, continuous infusion, and liposomal delivery of doxorubicin to tumor cells. *Neoplasia*. **2**(4), 325–338 (2000).

[24] Wenbo Zhan, [Mathematical modelling of drug delivery to solid tumour](https://core.ac.uk/download/pdf/76995866.pdf), PhD Thesis, Imperial College London (2014).

[25] Stylianopoulos, T., Economides, E.A., Baish, J.W., Fukumura, D., & Jain, R.K. Towards optimal design of cancer nanomedicines: multi-stage nanoparticles for the treatment of solid tumors. *Ann. Biomed. Eng.* **43**, 2291–2300 (2015).

[26] Kerr, D.J., Kerr, A.M., Freshney, R.I., & Kaye, S.B. Delivery of molecular and cellular medicine to solid tumors. *Biochem Pharmacol*. **35**, 12817–2823 (1986).

[27] Ebrahimi Zade, A., Shahabi Haghighi, H., & Soltani, M. Reinforcement learning for optimal scheduling of Glioblastoma treatment with Temozolomide. *Comput Methods Programs Biomed.* **193**,105443 (2020).

[28] Chou, C.Y., Chang, W.I., Horng, T.L., & Lin, W.L. Numerical modeling of nanodrug distribution in tumors with heterogeneous vasculature. *PLoS* *ONE*. **12**, e0189802 (2017).

[29] Zhang, A., Mi, X., Yang, G., Xu, & L.X. Numerical study of thermally targeted liposomal drug delivery in tumor. *Journal of Heat Transfer*. **131** (4), 043209 (2009).

[30] Ganz, D.E., Sexton-Stallone, B., Brackett, E.L., & Forbes, N.S. Tissue transport affects how treatment scheduling increases the efficacy of chemotherapeutic drugs. *J. Theor. Biol.* **438**, 21–33 (2018).

[31] Wang, Z. H. *et al*. Theory and experimental validation of a spatio-temporal model of chemotherapy transport to enhance tumor cell kill. *PLoS Comput*. *Biol*. **12**, e1004969 (2016).

[32] Brocato, T.A., Coker, E.N., Durfee, P.N. *et al*. Understanding the Connection between Nanoparticle Uptake and Cancer Treatment Efficacy using Mathematical Modeling. *Sci. Rep*. **8**, 7538 (2018).

[33] Pascal, J., Bearer, E.L., Wang, Z., Koay, E.J., Curley, S.A., Cristini, V., Mechanistic patient-specific predictive correlation of tumor drug response with microenvironment and perfusion measurements. *Proc Natl Acad Sci*. **110**(35), 14266–14271 (2013).

[34] Roudnicky, F., Yoon, S.Y., Poghosyan, S., Schwager, S., Poyet, C., Vella, G., Bachmann, S.B., Karaman, S., Shin, J.W., Otto, V.I., & Detmar, M. Alternative transcription of a shorter, non-anti-angiogenic thrombospondin-2 variant in cancer-associated blood vessels. *Oncogene*. **37**(19), 2573–2585 (2018).

[35] Moradi Kashkooli, F., Soltani, M., & Momeni M.M. Computational modeling of drug delivery to solid tumors: A pilot study based on a real image, *J. Drug Deliv. Sci. Technol.*, **62**, 102347 (2021).

[36] Mpekris, F., Athanassios, S.A., Pirentis, P., & Stylianopoulos, T. Stress-mediated progression of solid tumors: effect of mechanical stress on tissue oxygenation, cancer cell proliferation, and drug delivery. *Biomechanics and Modeling in Mechanobiology*. **14**(6), 1391–1402 (2015).

[37] Baxter, L.T. & Jain, R.K. Transport of fluid and macromolecules in tumors. II. Role of heterogeneous perfusion and lymphatics. *Microvasc. Res.* **40**, 246–263 (1990).

[38] Soltani, M., Sefidgar, M., Bazmara, H., Casey, M.E., Subramaniam, R.M., Wahl, R.L., & Rahmim, A. Spatiotemporal distribution modeling of PET tracer uptake in solid tumors. *Annals of Nuclear Medicine*. **31**(2), 109–124 (2017).

[39] McDougall, S.R., Anderson, A.R., & Chaplain, M.A. Mathematical modelling of dynamic adaptive tumour-induced angiogenesis: clinical implications and therapeutic targeting strategies. *J. Theor. Biol.* **241**(3), 564–589 (2006).
